# Ecological momentary assessment and intervention in physical activity and well-being: affective reactions, social-cognitive factors, and behaviors as determinants of physical activity and exercise

**DOI:** 10.3389/fpsyg.2013.00916

**Published:** 2013-12-06

**Authors:** Wolfgang Schlicht, Ulrich W. Ebner-Priemer, Martina Kanning

**Affiliations:** ^1^Department of Exercise Sciences and Health Science, University of StuttgartStuttgart, Germany; ^2^Karlsruhe Institute of TechnologyKarlsruhe, Germany; ^3^Central Institute of Mental HealthMannheim, Germany

**Keywords:** ambulatory assessment, ambulatory monitoring, ecological momentary assessment, experience sampling method, computer-assisted diary, ecological validity, accelerometry

Psychological research uses multifarious assessment strategies to describe and to explain emotional and cognitive processes accompanying behavior in order to predict future behavior. However, laboratory assessments and questionnaire approaches dominate psychology, which has been criticized lately (Baumeister et al., 2007). In general, the appropriateness of a method or an instrument depends on the research questions. Observational methods are on the shortlist if behavior is the construct in focus. Questionnaires are appropriate where attitudes or personal traits should be described. Compared to traits, which are by definition stable in time and consistent across different situations, affective constructs like emotions, mood, and affective reactions tend to be highly volatile. Affective dynamics are caused by internal and external influences of high variance, e.g., by bad or good news, by hormonal variations, etc. (Schlicht et al., 2013). Questionnaires to investigate affective states presume too much regarding subjects' power of memory. As different authors point out, memory processes are open to biases and distortions (e.g., Kahneman et al., 2004).

This is the same if subjects are asked to remember their volume of physical activity. The time, which one has invested in a sports game, in a tennis match or walking in a group or in other similar types of structured physical activity is often easy to remember. Questionnaires might be appropriate instruments to get reliable data in these situations. This is not the case with exercise bouts and with low intense physical activity like non-exercise thermogenesis, activities of daily living or all kinds of activities done during the course of a normal day. Only accelerometers or other types of electronic monitors registering data (e.g., bio data such as heart rate) will deliver valid and reliable information.

Another methodological question is raised if research seeks to gain data out of subjects' real life. If the ecological validity should be convincing research has to go into everyday life using a suitable approach to get valid and reliable data. Ambulatory Assessment (AA) or ecological momentary assessment is an appropriate and promising approach for infield real time investigations. The *Society for Ambulatory Assessment—Understanding Behavior in Context* defines AA as any use of “… infield methods to assess ongoing behavior, physiology, experience, and environmental aspects of humans …” (http://www.ambulatory-assessment.org; last access 03. October 2013).

Meanwhile, AA has gained increasing interest in psychology, has been used in countless studies and reviews, and several special issues have been published (e.g., in *European Psychologist edited by* Ebner-Priemer et al. (2009); in *Psychological Assessment* edited by Trull and Ebner-Priemer (2009); and in *Psychosomatice Medicine*, edited by Kubiak and Stone (2012). An internet search for AA delivers 11,100,000 entries and one article in Wikipedia. One can get the impression that those activities prove that AA is nowadays a common method in psychology, but it isn't. AA is still an approach needing special expertise. Real time data collection requires specialized hard- and software and that resulting longitudinal data needs sophisticated statistical analyses.

Devices for AA are computer-aided and allow for the collection of a huge variety of interesting parameters in daily life (for more details see Fahrenberg et al., 2002):

- self reported data (whereabouts, behaviors, settings);
- moods, affective reactions, symptoms like aches, commentaries;
- psychological tests infield;
- behaviors (e.g., physical activity; speech patterns);
- environmental conditions (e.g., noise, temperature);
- bio-markers (e.g., blood pressure, heart rate, skin response).

Although electronic diaries have gained increasing interest in psychology to assess variables of interest in real time, and although accelerometers are quite popular in sports and exercise science to objectively assess physical activity and movement pattern in everyday life, the combination of both methods is still alarmingly rare. To give researchers, in the context of physical activity and exercise psychology, guidance in the advantages and benefits of combining e-dairies with accelerometers, we invited experts to report their original studies. It resulted in a reader giving an impression of the fruitfulness of AA in this special field.

The reader starts with a position statement done by the editors themselves (Kanning et al., 2013), making suggestions “how to investigate within-subject associations.”

This statement is followed by an article written by Bossmann et al. (2013) looking for the association between short periods of everyday activity and mood. The authors examined the influence of various everyday life activities on three dimensions of mood (valence, calmness, energetic arousal) in a predominantly inactive sample.

Bussmann (2013), the next author in the reader, provokes with his thesis that “One plus one equals three (or more…).” He is convinced that “the time is right to combine advanced methods of measuring movement behavior with advanced use of e-diaries/e-questionnaires” resulting not just in adding the values of both separate fields, but surpassing this sum.

The same author, together with his co-author (Bussmann and van den Berg-Emons, 2013), discusses what can be measured beyond the total amount of activity. Contrary to health sciences where the volume or amount of physical activity is the crucial variable for determining the effect of this behavior in reducing the incidence of non-communicable diseases, Bussmann and his colleagues make the point that, when focussing other research questions, it is worth looking closer to single components of this multi-dimensional construct and to answer the methodological challenging question of which parameters are most relevant, valid and responsive in a given setting.

An original research article done by Dunton et al. (2012) follows this *theory article*. They tested the feasibility and validity of an EMA self-report protocol using electronic surveys on mobile phones. It is feasible and data are valid, but the volume of physical activity is underestimated, especially for those persons underweight or normal weight.

Ebner-Priemer et al. (2013) highlight the problem that physical activity can be monitored continuously, whereas psychological variables can only be assessed at discrete intervals. The challenge is to link both types of variables. The authors propose an interactive multimodal ambulatory monitoring algorithm, which automatically increases the number of e-diary assessments during “active” episodes.

Kanning (2013) uses objective, real-time measures to investigate the effect of actual physical activity on affective states in everyday life. In her original research article she reports a study differentiating the contexts of working and leisure time in a sample with students. There is an interesting moderator effect identified by multilevel analyses: Active episodes of physical activity and the context influenced subsequent energetic arousal. Valence and calmness seemed to be independent of the context in which the activity occurs.

From work and leisure time the next text goes to pupils in elementary schools. Kühnhausen et al. (2013) asked if and how it is possible to collect valid and reliable data in this special group of very young participants. It is feasible to objectively measure children's activity using accelerometers for a period of several weeks. In this investigation an impact of physical activity on affect in children was not shown.

Murphy et al. (2012) original research focuses on another group of participants; those with chronic pain. They investigate the association between symptoms, pain coping strategies and physical activity among patients with symptomatic knee and hip osteoarthritis. The higher the body mass index, subjective feelings of fatigue, and using “guarding” as coping method, the lower the activity levels have been. “Asking for assistance,” another coping method was related to higher activity levels.

Schwerdtfeger et al. (2012) report an ecological 108 momentary intervention study using a randomized experimental design. In the treatment group they used text messages to increase physical activity and to induce breaks of sedentary time in contrast to a group only having an educational standard intervention and an untreated control group. It is perhaps against our expectations, but short text messages reminding subjects of their action plans are not more effective than an intervention without text messages. However, it is comforting to see that there seems to be a beneficial effect on subject's self-efficacy and, as is known, this is a very strong predictor of behavior change.

Two articles in which von Haaren is a contributor (von Haaren et al., 2013; and Walter et al., 2013) close the series of articles. The second to last original research article done by von Haaren launches an interesting debate. Following their results, they state that analysing the physical activity and affect association of inactive people is difficult due to little variance and distribution of the assessed variables. In the last article in this reader Walter et al. found non-statistically significant increases in mood intensity immediately after acute endurance exercise episodes. Perhaps surprisingly, no medium term effects in mood states could be observed after a few weeks of endurance training, too.

Given the combination of texts the reader has a colorful bouquet of different inquiries in his hands and we hope this launches more studies in the natural environment assessing behavior in real settings.

## References

[B1a] Baumeister R. F. Vohs K. D. Funder D. C. (2007). Psychology as the science of self reports and finger movements: whatever happened to actual behavior? Perspect. Psychol. Sci. 2, 396–403 10.1111/j.1745-6916.2007.00051.x 26151975

[B1] Bossmann T. Kanning M. Koudela-Hamila S. Hey S. Ebner-Priemer U. (2013). The association between short periods of everyday life activities and affective states: a replication study using ambulatory assessment. Front. Psychol. 4:102 10.3389/fpsyg.2013.00102 23596426 PMC3625722

[B2] Bussmann J. B. (2013). One plus one equals three (or more …): combining the assessment of movement behavior and subjective states in everyday life. Front. Psychol. 4:216 10.3389/fpsyg.2013.00216 23720644 PMC3655276

[B3] Bussmann J. B. J. van den Berg-Emons R. J. G. (2013). To total amount of activity….. and beyond: perspectives on measuring physical behavior. Front. Psychol. 4:463 10.3389/fpsyg.2013.00463 23885248 PMC3717476

[B4] Dunton G. F. Liao Y. Kawabata K. Intille S. (2012). Momentary assessment of adults' physical activity and sedentary behavior: feasibility and validity. Front. Psychol. 3:260 10.3389/fpsyg.2012.00260 22866046 PMC3408114

[B5] Ebner-Priemer U. Kubiak T. Pawlik K. (2009). Ambulatory Assessment. Eur. Psychol. 14, 95–97 10.1027/1016-9040.14.2.109

[B6] Ebner-Priemer U. W. Koudela S. Mutz G. Kanning M. (2013). Interactive multimodal ambulatory monitoring to investigate the association between physical activity and affect. Front. Psychol. 3:596 10.3389/fpsyg.2012.00596 23355828 PMC3554220

[B7] Fahrenberg J. Leonhart R. Foerster F. (2002). Alltagsnahe Psychologie mit Hand-Held PC und Physiologischem Mess-System. Bern: Huber

[B8] Kahneman D. Krueger A. B. Schkade D. A. Schwarz N. Stone A. A. (2004). A survey method for characterizing daily life experience: the day reconstruction-method. Science 306, 1776–1780 10.1126/science.1103572 15576620

[B9] Kanning M. (2013). Using objective, real-time measures to investigate the effect of actual physical activity on affective states in everyday life differentiating the contexts of working and leisure time in a sample with students. Front. Psychol. 3:602 10.3389/fpsyg.2012.00602 23346064 PMC3549542

[B10] Kanning M. K. Ebner-Priemer U. W. Schlicht W. M. (2013). How to investigate within-subject associations between physical activity and momentary affective states in everyday life: a position statement based on a literature overview. Front. Psychol. 4:187 10.3389/fpsyg.2013.00187 23641221 PMC3638123

[B11] Kubiak T. Stone A. (2012). Ambulatory monitoring of biobehavioral processes in health and disease. Psychosom. Med. 74, 325–326 10.1097/PSY.0b013e31825878da 22582329

[B12] Kühnhausen J. Leonhardt A. Dirk J. Schmiedek F. (2013). Physical activity and affect in elementary school children's daily lives. Front. Psychol. 4:456 10.3389/fpsyg.2013.00456 23885246 PMC3717478

[B13] Murphy S. L. Kratz A. L. Williams D. A. Geisser M. E. (2012). The association between symptoms, pain coping strategies, and physical activity among people with symptomatic knee and hip osteoarthritis. Front. Psychol. 3:326 10.3389/fpsyg.2012.00326 22969747 PMC3432514

[B14] Schlicht W. Reicherz A. Kanning M. (2013). Affecive reactions to physical activity, exercise and the activities of daily living: a review, in Handbook of Psychology of Emotions, eds Mohiyeddini C. Eysenck M. Bauer S. (New York, NY: Nova Science), 143, 307–324

[B15] Schwerdtfeger A. R. Schmitz C. Warken M. (2012). Using text messages to bridge the intention-behavior gap? A pilot study on the use of text message reminders to increase objectively assessed physical activity in daily life. Front. Psychol. 3:270 10.3389/fpsyg.2012.00270 22876237 PMC3410409

[B16] Trull T. J. Ebner-Priemer U. (2009). Psychopathology in daily life: using ecological momentary assessment methods. Psychol. Assess. 21, 457 10.1037/a0017075 19947781

[B17] von Haaren B. Loeffler S. N. Haertel S. Anastasopoulou P. Stumpp J. Hey S. (2013). Characteristics of the activity-affect association in inactive people: an ambulatory assessment study in daily life. Front. Psychol. 4:163 10.3389/fpsyg.2013.00163 23580167 PMC3619104

[B18] Walter K. von Haaren B. Löffler S. Härtel S. Jansen C.-P. Werner C. (2013). Acute and medium term effects of a 10-week running intervention on mood state in apprentices. Front. Psychol. 4:411 10.3389/fpsyg.2013.00411 23847579 PMC3705169

